# Fingertip rapid point-of-care test in adult case-finding in coeliac disease

**DOI:** 10.1186/1471-230X-13-115

**Published:** 2013-07-12

**Authors:** Alina Popp, Mariana Jinga, Ciprian Jurcut, Vasile Balaban, Catalina Bardas, Kaija Laurila, Florina Vasilescu, Adina Ene, Ioana Anca, Markku Mäki

**Affiliations:** 1 University of Medicine and Pharmacy “Carol Davila”, Str. Dionisie Lupu nr. 37, sector 1, 020022 Bucharest, Romania; 2 Institute for Mother and Child Care “Alfred Rusescu”, Bdv. Lacul Tei nr. 120, sector 2, 020395 Bucharest, Romania; 3 Central University Emergency Military Hospital “Dr. Carol Davila”, Str. Mircea Vulcanescu nr. 88, sector 1, 010825 Bucharest, Romania; 4 Tampere Center for Child Health Research, University of Tampere and Tampere University Hospital, Finn-Medi 3, Biokatu 10, 33520 Tampere, Finland

**Keywords:** Coeliac disease, Healthy relatives, Case-finding, Point-of-care test

## Abstract

**Background:**

Coeliac disease (CD), due to its protean clinical manifestation, is still very under diagnosed in adults and delays in diagnosis may take years and even decades. Simple tools to find cases in primary care may help to identify patients for further diagnostic tests. We have evaluated the usefulness of an on site rapid fingertip whole blood point-of-care test (POCT) for such a purpose.

**Methods:**

As CD is known to run within families, we tested 148 healthy relatives of 70 Romanian index cases with biopsy-proven CD (87% of all first-degree family members, median age 36 years) for the presence of circulating autoantibodies. In addition to performing the POCT (which measures blood erythrocyte self-TG2-autoantibody complexes) on site, blood was drawn for later evaluations of serum IgA-class endomysial antibodies (EMA). EMA-positive sera were further tested for transglutaminase 2 antibodies (TG2-IgA). All serological parameters were analyzed blindly in a centralized laboratory that had no knowledge of the on site POCT result. Endoscopic small intestinal biopsies was recommended for all POCT- or EMA-test positive subjects.

**Results:**

In on site testing the POCT was positive in 12/148 first-degree relatives (8%) and all these subjects were also serum EMA-positive. A positive EMA test was found only in one other subject. All remaining 135 healthy first-degree relatives were negative for both POCT and EMA. Four subjects positive for both POCT and EMA were negative for TG2-IgA. Ten out of thirteen of the antibody-positive subjects agreed to undergo endoscopy. The POCT was found to be positive in 8/9 first-degree relatives having coeliac-type mucosal lesions of grade Marsh 2 (n = 3) or Marsh 3 (n = 6). The three POCT-positive subjects not agreeing to undergo endoscopy were also both EMA- and TG2-IgA-positive.

**Conclusion:**

The fingertip whole blood rapid POCT might fulfill the unmet need for a simple and cheap case-finding biomarker for early detection and presumptive diagnosis of CD. Confirmatory studies are warranted in adult case-finding in specialized outpatient clinics and in primary care.

## Background

Coeliac disease (CD) is a systemic disorder triggered by the ingestion of gluten, a protein present in wheat, rye and barley. Gluten injures the small intestinal mucosal lining leading to villous atrophy and crypt hyperplasia [1]. Individuals affected by the disease also share the autoimmune extended human leukocyte antigen (HLA) haplotypes DR3-DQ2, DR11/7-DQ2 or DR4-DQ8. The prevalence of CD in various populations is around 1% [2,3] but there are countries with lower and higher prevalence [4]. There is also evidence that the disease itself is increasing over time, similar to other autoimmune diseases in general [5-8]. CD manifests at all ages. It may be clinically silent, patients may show only vague symptoms or present with extraintestinal problems including psychiatric, neurologic, bone, liver or reproductive disorders or as malignancy [9-12].

It is well known that CD runs within families, and first-degree relatives of patients are at risk of developing the disorder [13-15]. By reason of the protean manifestation of CD, clinical suspicion is not obvious, and it is clear that this disorder is highly under diagnosed worldwide [1,4,7,16]. Even if health professionals are aware of diagnostic delays, this period of latency persists and is counted in years and decades [17-19]. Screen-detected patients with clinically silent disease but manifest small-intestinal mucosal lesion seem to benefit from a gluten-free diet [20-22]. It has in fact been claimed that CD detected by screening is not silent but simply unrecognized [23]. Also new CD management models include first-degree relatives as a high-risk group and recommend case-finding screening within families [24].

Active case-finding in primary care using sensitive and specific biomarkers of CD might be a solution to shorten diagnostic delays. Centralized laboratories provide autoantibody tests such as endomysial (EMA) and antitransglutaminase 2 (TG2) IgA antibody tests [25,26]. However, centralized laboratory tests are not easily available or affordable for medical health care in many parts of the world. An easy-to-use on site whole blood self-TG2-based fingertip point-of-care test (POCT) has been shown to be effective in CD case finding in schoolchildren at a population-based level and in children with type 1 diabetes [21,27]. The POCT proved to be highly sensitive and specific resulting in an accuracy of >95% to detect untreated CD [28]. Also, a recent evidence report showed the POCT to have a high pooled sensitivity and specificity in case finding of CD [26]. We hypothesize that a less invasive and less expensive POCT biomarker is able to pick out clinically silent CD in adults in a low-prevalence population. To test this hypothesis we chose to study CD families where the disease would be expected to be prevalent among first-degree relatives.

## Methods

### Patients

170 first-degree healthy relatives of 70 index cases (77 children and 15 adults) were invited over a five years period (2007–2012) to be evaluated for the presence of coeliac-autoantibodies. The inclusion criterion for the index cases was biopsy-proven CD (Marsh 3 mucosal lesion). Of the 170 subjects, 148 (18 children, 130 adults; median age 36 years, range 1.2-77) agreed to participate. The remaining 22 could not been reached or declined. At the time of the evaluation, all participants confirmed that they were consuming a regular gluten-containing diet.

### Whole blood and serum case-finding biomarkers

The screening for the presence of CD autoantibodies in the healthy relatives took place on the same day as the diagnostic examinations of the index patients or in certain cases later, during regular follow-up visits. All centralized laboratory serologic determinations were made in blinded fashion without knowledge of the on-site fingertip whole blood POCT results.

The Biocard Celiac Test, AniBiotech, Vantaa, Finland, was selected as the point-of-care biomarker test for CD case-finding. This rapid whole blood self TG2-based IgA-class fingertip test uses the patient’s own endogenous TG2 found in the erythrocytes of a whole blood sample. When the finger-tip blood is haemolyzed, the liberated TG2 forms a complex with circulating autoantibodies, if present in the same sample. Blood erythrocyte self-TG2-autoantibody complexes are captured from the haemolyzed sample by TG2-binding proteins onto a solid phase and the presence of autoantibodies is measured by labeled antihuman IgA [28]. The results of the POCT were evaluated visually on site after five minutes, but no later than ten minutes, according to the manufacturer’s recommendations (line visible in test window – positive result; no line in test window – negative result).

On the same day when the POCT was performed, venous blood was drawn for laboratory serum autoantibody measurements. Serum IgA-class antiendomysial antibodies (EMA) were determined using an indirect immunofluorescence method (Nova Lite, Inova Diagnostics, CA, USA) with cut-off for positivity at a serum dilution of 1:5. Further serum dilutions (1:50, 1:100, 1:200, 1:500, 1:1000, 1:2000, 1:4000) were made for initial positive samples till the highest positive titer was obtained for each sample. In the present series, no cases of selective IgA deficiency were found.

Serum samples showing positivity in the EMA test were further evaluated for TG2-IgA (Celikey, Phadia GmbH, Freiburg, Germany) using an enzyme-linked immunosorbent assay (ELISA) with a positivity cut-off at 5 U/ml, as recommended by the product manufacturer.

### Endoscopy and biopsies

Upper gastrointestinal endoscopy with small-bowel mucosal biopsies was recommended for all subjects with positive POCT or EMA tests. During endoscopy, up to 5 duodenal mucosal specimens were taken. The biopsy specimens were fixed in formalin, embedded in paraffin, cut, stained with haematoxylin-eosin and scored by pathologists (AE and FV) according to the Marsh-Oberhuber classification (Marsh 0, 1, 2, 3a, 3b, or 3c) [29,30].

### Ethics approval

The study was approved by the Ethical Committee of the University and Pharmacy “Carol Davila” and Ethical Committee of the Institute for Mother and Child Care “Alfred Rusescu” Bucharest, Romania. The antibody-positive subjects or their parents gave written informed consent for upper gastrointestinal endoscopy.

## Results

In total, 12 out of 148 (8%) first-degree healthy relatives showed positivity in the POCT. All POCT-positive subjects proved to be EMA-positive, giving 100% specificity for the POCT against the reference standard for serum CD autoantibody [24]. Only one more positive subject was found by the EMA test (Table 1) and all the remaining 135 healthy first-degree relatives were negative both in the POCT and for EMA.

**Table 1:** Spectrum of coeliac type serum autoantibodies and small-intestinal mucosal biopsy outcome in healthy first-degree relatives of coeliac disease (CD) patients

**Case no.**	**Gender**	**Age at CD antibody evaluation (years)**	**POCT**	**EMA-IgA (serum dilution step)**	**TG2-IgA (U/ml)**	**Biopsy outcome (Marsh classification)**
1	F	39	+	1:4000	821.5	3c
2	F	38	+	1:200	37.4	NA
3	F	36	+	1:1000	61.8	NA
4	F	34	+	1:5	2.3	3b
5	F	40	+	1:2000	499.4	NA
6	F	42	+	1:200	25.6	1
7	F	56	-	1:50	4.2	2
8	F	2.4	+	1:50	3.4	3a
9	M	10.7	+	1:5	3.5	2
10	F	36	+	1:50	4.0	3a
11	F	46	+	1:4000	40.2	3c
12	F	31	+	1:200	21.4	2
13	F	2	+	1:1000	72.8	3c

Abbreviations: *F* female, *M* male, *NA* not available, *POCT* point of care test, *EMA* antiendomysial antibody, *TG2-IgA* IgA class transglutaminase 2 antibodies.

Altogether 10/13 antibody positive family members were adults (median age 38.5 years). Four subjects positive for both POCT and EMA were negative for TG2-IgA when using the manufacturer’s positivity cut-off of 5 U/ml. When again using 3 U/ml as cut-off, as suggested by the new ESPGHAN 2012 criteria [24], only one EMA-positive subject would have been missed by the ELISA method. Her EMA titre was low, 1:5, but she was also POCT-positive (case 4 in Table 1). The only EMA-positive subject, case 7 in Table 1, with a low serum titer of 1:50 and yielding a negative test result in POCT, had a TG2-IgA titer of 4.2 U/ml.

Ten of the thirteen antibody-positive first-degree relatives agreed to undergo upper gastrointestinal endoscopy and duodenal biopsies. All but one showed coeliac-type mucosal lesions of grade Marsh 2 (n = 3) and Marsh 3 (n = 6), as summarized in Table 1. POCT was found to be positive in eight of the nine subjects with biopsy-proven CD. The three POCT-positive subjects not agreeing to undergo endoscopy also showed marked positivity in both EMA and TG2-IgA tests (Table 1). The prevalence of coeliac gluten sensitivity was 8.7% as determined by positive autoantibodies.

## Discussion

The present study emphasizes the usefulness of a simple rapid fingertip whole blood POCT for a presumptive diagnosis of CD. The test was able to select correctly individuals, even clinically silent ones, for further confirmatory diagnostic studies such as duodenal biopsies. We show that the POCT tested on site worked hand in hand with the centralized laboratory golden standard CD autoantibody testing, the EMA test [24]; among 148 tested subjects there was only one discrepant result, a POCT-negative person who had a low serum EMA titer but who was also negative for TG2-IgA (case No. 7 in Table 1). Furthermore, gluten-induced small intestinal mucosal lesion, i.e. Marsh class 2 and 3, was detected in all but one of the biopsied subjects. From this series of individuals we also noted that even low titers of EMA positivity may be indicative of mucosal injury.

To confirm or disprove our hypothesis that the used POCT biomarker is able to pick out CD also in adults, we chose to study first-degree family members of CD patients. In fact, 77% of the family members positive for the test were adults and our results are in agreement with those previously obtained in children [21,27,28]. The present POCT results also confirm the results of a recent report where POCT was shown to have a pooled sensitivity of 96.4% and pooled specificity of 97.7% for detection of CD [26]. The same report showed the centralized hospital ELISA TG2-IgA in 11/15 studies to have a sensitivity of >90% and in 13/15 studies a specificity of >90%. ELISA testing for the presence of TG2-IgA seems to lead to false-positive results especially in individuals carrying a high risk of CD such as relatives of patients [31]. As reviewed by Husby et al. [24], TG2-IgA positivity without CD is also seen in autoimmune disorders, in patients with infections, tumors, psoriasis and heart and liver diseases. The epitopes in TG2 for the false-positive antibodies in other diseases and seen in ELISA tests differ from the TG2 epitopes which CD patient autoantibodies target. Further, positive TG2-IgA ELISA results in patients without CD do not correlate with the EMA results [32].

On-site use of the POCT seems to provide a strong tool for the health care system in case-finding of CD. This offers immediate results and potentially shortens the diagnostic process in adults [17-19]. Moreover, the use of a POCT in primary care might lower the costs related to equipment, trained personnel and time-management of centralized serological tests. As of now, a small-intestinal biopsy is still needed for confirmation of diagnosis. But on the other hand, the present study results support the new global guidelines on CD stating that TG2-IgA can be assessed in the physician’s office by using the self antibody-based rapid finger-tip test, and in areas with limited resources a rapid test may be used as sole diagnostic means for CD [33].

A limitation in the present study is the relatively small population studied. The study should be repeated in larger adult patient populations in primary care and specialized outpatient clinics dealing with patients with vague and various symptoms or signs where CD could be the underlying cause of ill health.

The serologic prevalence of CD in our study in Romania was 8.7%, similar to that reported in other family studies [13-15]. The present results argue, similarly to a recent case-finding study in type 1 diabetes [27], that the CD prevalence in Romania is probably much higher than previously thought. In fact, there has previously been an indication that the disease exists in adults in Romania, unspecific symptoms have shown a frequent small-intestinal mucosal injury indicative of CD [34]. Overall, the POCT seems to work as well as the EMA test in case identification [14]. A recent Austrian study indicates that the POCT is a reliable, easy to use and well-accepted tool even for home testing of first- and second-degree relatives of patients with CD [35].

## Conclusion

In conclusion, case-finding in the primary care using a fingertip whole blood on site POCT could be the way forward in the early detection and diagnosis of celiac disease.

## Abbreviations

CD: Coeliac disease; POCT: Point-of-care test; EMA: Endomysial antibodies; TG2-IgA: Transglutaminase 2 antibodies; HLA: Human leukocyte antigen; ELISA: Enzyme-linked immunosorbent assay.

## Competing interests

Ani Biotech, who donated the point of care test kits, did not take part in designing or executing the study, were not involved in reading the results or in writing the manuscript. MM is one inventor of the patent “Methods and Means for Detecting Gluten-Induced Diseases”, USA States Patent Number 7,361,480 - USA, Patent Granted 22.4.2008; European Patent No. 1390753, European Patent Office 22.10.2008. Finn Medi Oy Ltd, owned by the University of Tampere and Tampere University Hospital, Finland, has commercialized the innovation and licensed it to Ani Biotech, which used it to develop the Biocard Celiac Test. Finn Medi and Ani Biotech were not involved in the study. The other authors declare no competing interests.

## Authors’ contributions

AP contributed to the conception and design, supervised all aspects of the evaluation, carried out the collection and analysis of data, wrote the initial version of the manuscript and approved the final manuscript as submitted. MJ provided supervision in all aspects of the evaluations, including design, clinical examination, endoscopic biopsies critically revised the manuscript and approved the final version as submitted. CJ contributed to the literature search and analysis of data, clinical management of adult participants, critically revised the manuscript and approved the final version as submitted. VB made a substantial contribution to data collection and analysis, assisted with the initial manuscript preparation and approved the final version as submitted. CB made a substantial contribution to all aspects of evaluation, including clinical examination and management of pediatric participants, acquisition and analysis of data, revised the manuscript and approved the final text as submitted. AE and FV made a contribution on the interpretation of data, provided histological interpretation of the mucosal biopsies, critically revised the manuscript and approved the final text as submitted. KL contributed substantially to the data results, revised the manuscript and approved the final version as submitted. IA contributed substantially to the design and interpretation of data, critically revised the manuscript for important intellectual content and approved the final manuscript as submitted. MM is the senior author and expert in the field; he was responsible for the conception, design and the interpretation of data, contributed to obtaining of funding, made a substantial contribution to the manuscript preparation, including review, critique and advice on each version of the manuscript, directed the literature search and approved the final version of the manuscript as submitted.

## Pre-publication history

The pre-publication history for this paper can be accessed here:

http://www.biomedcentral.com/1471-230X/13/115/prepub
